# Sensitive stripping voltammetric determination of an anthracene–isatin Schiff base in biological samples

**DOI:** 10.55730/1300-0527.3791

**Published:** 2026-03-11

**Authors:** Gökçe ÖZTÜRK, İrem GÖKGÖZ, Emine ARSLAN, Elif ŞİŞMAN, İnci Selin DOĞAN, Dilek KUL, Fatma AĞIN

**Affiliations:** 1 Department of Analytical Chemistry, Faculty of Pharmacy, Karadeniz Technical University, Trabzon, Turkiye; 2 Department of Analytical Chemistry, Graduate School of Health Sciences, Karadeniz Technical University, Trabzon, Turkiye; 3 Department of Pharmaceutical Chemistry, Faculty of Pharmacy, Karadeniz Technical University, Trabzon, Turkiye

**Keywords:** Adsorptive stripping voltammetry, electrochemical oxidation, glassy carbon electrode, Schiff base, serum analysis

## Abstract

In the present study, the electrochemical behavior and quantitative determination of the Schiff base 3-[(anthracen-2-yl)imino]-5-nitro-1,3-dihydro-2H-indol-2-one (ANT) were investigated for the first time using voltammetric techniques at a glassy carbon electrode. The effects of solvent composition, pH, and scan rate on the redox behavior of ANT were systematically evaluated. The compound exhibited an irreversible oxidation process governed by adsorption-controlled kinetics. Quantitative analysis was conducted using differential pulse stripping voltammetry (AdS-DPV) and square-wave stripping voltammetry (AdS-SWV) under optimized conditions. Both methods exhibited linear responses over the concentration range of 0.2–8.0 μM, with limits of detection of 0.0366 μM and 0.0433 μM for AdS-DPV and AdS-SWV, respectively. The precision of the methods was confirmed through intra- and interday measurements, with relative standard deviations below 1.73%. Recovery studies performed in protein-free human serum yielded 99.78% and 99.52% recoveries for AdS-DPV and AdS-SWV, respectively, demonstrating their applicability to biological matrices. Mechanistic studies using 5-nitroisatin and indomethacin as model compounds indicated that ANT predominantly undergoes oxidation at the anthracene core, an outcome influenced by the nitro group, rather than at the isatin moiety. Collectively, the results reveal the developed voltammetric procedures to be simple, selective, sensitive, and reproducible, providing robust and reliable tools for the electrochemical analysis of this anthracene-isatin Schiff base derivative in biological matrices.

## 1. Introduction

Schiff bases constitute an important class of compounds formed by the condensation of primary amines with aldehydes or ketones, and are characterized by the azomethine (C=N) functional group. Owing to their structural diversity, they have attracted considerable attention in the pharmaceutical and coordination chemistry fields, as well as in materials science. There have been numerous studies to date documenting their broad pharmacological activities, which include antimicrobial, anticancer, antifungal, antioxidant, and anti-inflammatory effects [1–6]. Their pronounced ability to form stable complexes with transition-metal ions further expands their applications in catalysis, sensing, and bioinorganic chemistry [7].

Indole and isatin derivatives are widely employed as building blocks in Schiff base synthesis due to their significant biological relevance. Indole is a privileged heterocycle found in biomolecules such as tryptophan and serotonin, and its derivatives exhibit diverse therapeutic properties [8]. Similarly, isatin derivatives demonstrate notable antimicrobial, anticancer, and antioxidant activity. Despite the wealth of research on the biological properties of indole- and isatin-based Schiff bases, their electrochemical characteristics remain relatively underexplored. A detailed investigation of their redox behavior is essential for understanding structure–activity relationships, predicting metabolic transformation pathways, and designing new voltammetric sensing platforms.

Anthracene derivatives constitute another important class of compounds whose extended π-conjugated systems give rise to unique photophysical, optoelectronic, and biological properties [9–11]. Schiff bases incorporating anthracene moieties have been investigated for their enhanced electron-transfer characteristics and potential applications in optical and sensing technologies. Analytical and spectral studies of anthracene-derived Schiff ligands and their metal complexes have demonstrated the importance of conjugated aromatic frameworks in facilitating electron transfer processes [12]. Recent theoretical and experimental research has further clarified the distinctive photophysical and electrochemical behavior of anthracene-based Schiff bases [13,14]. However, systematic electrochemical investigations of Schiff bases derived from both anthracene and indole scaffolds remain scarce.

Powerful and versatile electrochemical tools have been developed to probe the redox properties of organic molecules. Cyclic voltammetry (CV) is widely employed to evaluate the redox reversibility, peak potentials, and mechanistic features of electron-transfer processes, whereas differential pulse voltammetry (DPV) and square-wave voltammetry (SWV) offer high sensitivity and low detection limits suitable for analytical applications [15]. Among the various electrode materials, glassy carbon electrodes (GCE) are generally preferred due to their broad potential window, chemical inertness, low background current, and excellent reproducibility [16]. Consequently, GCE-based voltammetric methods can be considered particularly effective for studying organic Schiff base derivatives.

In the present study, the Schiff base 3-[(anthracen-2-yl)imino]-5-nitro-1,3-dihydro-2H-indol-2-one (ANT) was electrochemically characterized for the first time at a GCE. A comprehensive investigation by CV, DPV, and SWV under various supporting electrolytes and pH conditions was conducted to elucidate its redox behavior and the number of electrons and protons involved in the oxidation process. The study presents a detailed electrochemical profile of ANT and a framework for the determination of the redox behavior of anthracene–indole Schiff bases. To the best of our knowledge, this is the first study to describe a reliable voltammetric approach to the determination of the electrochemical behavior of ANT.

## 2. Materials and methods

### 2.1. Instrumentation

Electrochemical measurements were performed using a μStat 300 bipotentiostat (Metrohm DropSens, Spain) controlled with DropView 8400 software. A conventional three-electrode configuration was employed, consisting of a GCE (BASi, 3 mm diameter) as the working electrode, a platinum wire counter electrode, and an Ag/AgCl (3.0 M KCl, BASi) reference electrode. Prior to each measurement, the GCE surface was manually polished on a BASi polishing pad with an alumina slurry (0.01 μm). All experiments were carried out at ambient temperature (25 ± 2 °C).

### 2.2. Reagents

ANT was synthesized according to a procedure previously reported by a coauthor of this study [17]. Briefly, 5-nitroisatin and the appropriate aromatic amine were dissolved in ethanol, and acetic acid was added as a catalyst. The reaction mixture was stirred at room temperature until completion. The resulting precipitate was filtered and purified by recrystallization from an ethanol/water (3:1, v/v) mixture. ANT was obtained as yellow crystals with a yield of 88% and was used in the electrochemical experiments without further purification. The structure and purity of the compound were confirmed by FT-IR, ^1^H NMR, ^13^C NMR, and LC–MS analyses.

A stock solution of ANT (1.0 × 10^−3^ M) was prepared in acetonitrile using a 10 mL volumetric flask. Working solutions were freshly prepared by diluting the stock solution with the appropriate supporting electrolyte, maintaining a constant acetonitrile content (10%, v/v).

Voltammetric measurements were performed in various supporting electrolytes, including 0.1 M sulfuric acid (H_2_SO_4_), 0.1 M phosphate buffer (PB, pH 6.0–8.0), 0.04 M Britton-Robinson buffer (BRB, pH 2.0–10.0), and 0.1 M acetate buffer (AcB, pH 3.5–5.5). Sterile-filtered human serum (male AB plasma; Sigma-Aldrich, USA) was stored at low temperature in the dark until use.

Solution pH values were measured and adjusted using an HI2211 pH meter (Hanna Instruments, Romania). All solutions were prepared using ultrapure water (resistivity ≥18.2 MΩ·cm) obtained from a Arium ProUV water purification system (Sartorius, Germany). Analytical-grade reagents (Merck or Sigma-Aldrich) were used without further purification.

### 2.3. Preparation of spiked human serum samples

A serum-based ANT stock solution (1.0 × 10^−3^ M) was prepared by mixing appropriate aliquots of human serum with the ANT stock that had been previously dissolved in acetonitrile. Serum proteins were precipitated by adding acetonitrile at a ratio of 1.5:1 (acetonitrile:serum, v/v). Both the ANT-spiked and blank serum samples were sonicated for 15 min and then centrifuged at 5000 rpm for 15 min to remove precipitated proteins. The clear supernatants were transferred to volumetric flasks and diluted with the selected supporting electrolyte to the desired volume, maintaining a final acetonitrile content of 10% (v/v) [18].

## 3. Results and discussion

### 3.1. Effect of solvent on the electrochemical behavior of ANT

The electrochemical behavior of ANT was first examined in various solvent systems to evaluate the effect of solvent polarity on its redox properties. As well-defined voltammetric signals could not be obtained due to the poor solubility of ANT in purely aqueous media, methanol, ethanol, and acetonitrile were tested as organic modifiers to improve solubility and voltammetric response.

Stock solutions of ANT (1.0 × 10^−3^ M) were prepared separately in methanol, ethanol, and acetonitrile. Working solutions (1.0 × 10^−4^ M) were then obtained by diluting these stocks into various supporting electrolytes, including H_2_SO_4_, BRB (pH 2.0, 3.0, and 9.0), AcB (pH 5.5), and PB (pH 7.0), while maintaining a constant organic solvent ratio of 10% (v/v). The electrochemical response of ANT in each medium was assessed using CV, DPV, and SWV.

Among the supporting electrolytes tested, solutions containing 10% acetonitrile yielded well-defined, symmetrical anodic peaks in both CV and DPV measurements. In contrast, media containing 10% methanol or ethanol consistently yielded broader, less symmetric peaks, often accompanied by distinct shoulders at more positive potentials. SWV analyses showed similar behavior, with methanol- and ethanol-containing solutions generating asymmetric peak shapes and additional oscillatory features. These results indicate that the enhanced voltammetric performance observed in acetonitrile is independent of the electrolyte composition. Acetonitrile was thus selected as the most suitable organic modifier for further electrochemical studies.

To determine the optimal acetonitrile content, AcB at pH 5.5 was selected from the tested supporting electrolytes, as it provided the highest and most symmetrical peak response. The effect of acetonitrile content (10%, 20%, 30%, and 40%, v/v) on the peak potential and peak current of ANT was investigated using working solutions (1.0 × 10^−4^ M) prepared in this buffer. CV, DPV, and SWV measurements were performed for each composition, and the peak current values obtained using the three techniques are presented in Table 1. The results showed that 10% acetonitrile produced the highest peak current in DPV. For CV, the peak current remained nearly constant between 10% and 30% acetonitrile, while SWV exhibited a slight increase at 20%. A decrease in peak current was observed at higher acetonitrile contents (30–40%) for all techniques, and increasing the acetonitrile percentage resulted in broader and less symmetrical peaks. Considering both peak current and peak quality, 10% acetonitrile provided the most well-defined and symmetrical voltammetric response and was therefore selected as the optimal medium for the voltammetric analysis of ANT.

### 3.2. Electrochemical behavior of ANT

**Figure 1:**
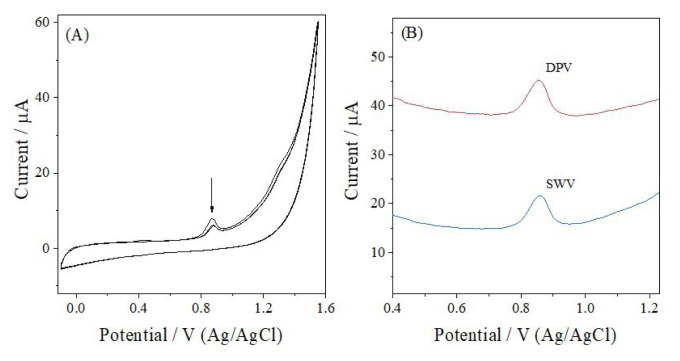
(A) CV and (B) DPV and SWV voltammograms of 6.0 × 10^−5^ M ANT obtained at a GCE in PB at pH 6.0.

Cyclic voltammetric measurements were performed using a GCE in 6.0 × 10^−5^ M ANT prepared in PB at pH 6.0 over the potential range from −0.3 V to +1.5 V (Figure 1A). The CV showed a single anodic peak at approximately +0.88 V with no corresponding cathodic peak, indicating an irreversible oxidation process. Peak current was noted to decrease progressively in successive scans, suggesting adsorption or accumulation of ANT or its oxidation products on the electrode surface, leading to partial surface passivation. The oxidation peak current decreased from 3.59 μA in the first scan to 2.15 μA and 1.94 μA in the second and third scans, corresponding to decreases of 40.1% and 45.9%, respectively. Figure 1B presents the DPV and SWV responses under the same conditions. Both techniques produced a single, well-defined anodic peak, indicating a clean, stable voltammetric response for ANT and supporting the behavior observed in CV.

### 3.3. Effect of pH on the electrochemical behavior of ANT

**Figure 2:**
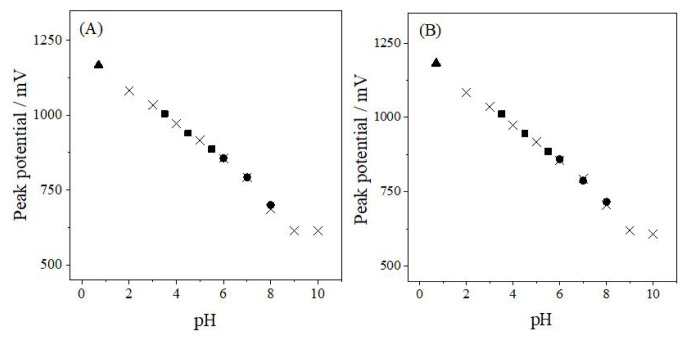
Effect of pH on the peak potential of ANT obtained using (A) DPV and (B) SWV. ▲: H_2_SO_4_, ×: BRB, ■: AcB, ●: PB

To determine the optimum pH and supporting electrolyte, solutions of 6.0 × 10^−5^ M ANT containing 10% (v/v) acetonitrile were prepared in different supporting media, and their electrochemical responses were evaluated using CV, DPV, and SWV. As can be seen in Figure 2, the anodic peak potential (*E*_p_) shifted linearly toward less positive values as pH increased, demonstrating the involvement of protons in the oxidation mechanism of ANT. This linear trend persisted up to pH 9.0, after which a clear deviation was observed. The inflection suggests a change in the protonation state of ANT or a modification in the electrode reaction pathway at higher pH values.

The *E*_p_–pH relationships obtained from DPV and SWV clearly demonstrate that ANT undergoes a coupled electron–proton transfer process during electrooxidation. The slopes calculated from the linear regions were 66.9 mV/pH for DPV and 65.5 mV/pH for SWV, both of which are in close agreement with the theoretical Nernstian value of 59 mV/pH. These findings support a mechanism involving equal numbers of protons and electrons in the rate-determining step, as reflected in the linear *E*_p_–pH correlations presented in equations 1 and 2 below.


(1) $$E_{p}(\text{mV})=1236.7-66.9\;\text{pH }(R^{2}=0.988,\text{pH }0.7-9.0,\;\text{for DPV})$$

(2) $$E_{p}(\text{mV})=1233.6-65.5\;\text{pH }(R^{2}=0.991,\text{pH }0.7-9.0,\;\text{for SWV})$$

**Figure 3:**
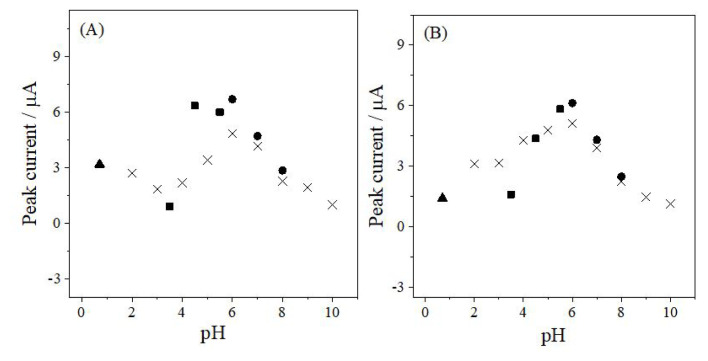
Effect of pH on the peak current of ANT obtained using (A) DPV and (B) SWV. ▲: H_2_SO_4_, ×: BRB, ■: AcB, ●: PB.

The peak current–pH profiles obtained from DPV and SWV (Figure 3) were examined to identify the most suitable conditions for quantitative analysis. Among the supporting media tested, PB at pH 6.0 provided both the highest peak currents and the most symmetrical peak shapes, and was therefore selected as the optimal medium for subsequent measurements.

### 3.4 Effect of scan rate on the electrochemical behavior of ANT

**Figure 4:**
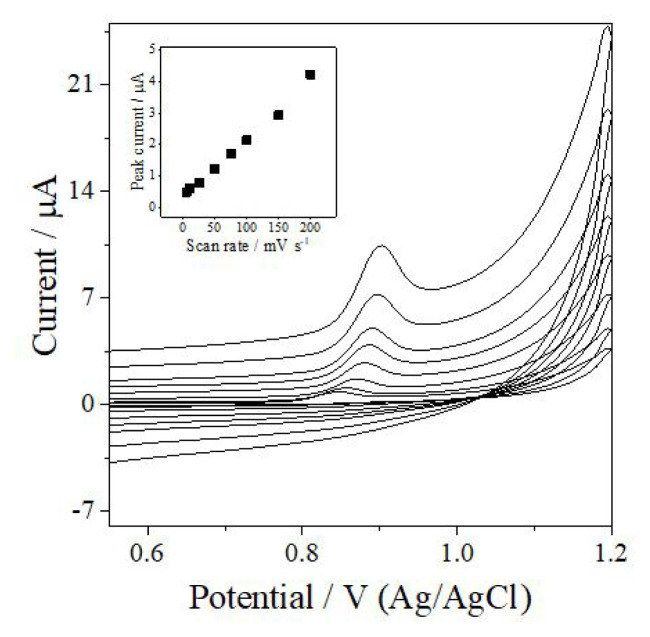
Cyclic voltammograms of 6.0 × 10^−5^ M ANT at a GCE in PB at pH 6.0 recorded at scan rates of 5, 10, 25, 50, 75, 100, 150, and 200 mV s^−1^. Inset: *I*_p_ -ν plot.

Scan rate (ν) studies were performed to gain insight into the kinetics and mechanism of ANT electrooxidation. Cyclic voltammograms were recorded for 6.0 × 10^−5^ M ANT in PB at pH 6.0 containing 10% acetonitrile over a scan rate range of 5–200 mV s^−1^ (Figure 4). As the scan rate increased, so did the anodic peak current (*I*_p_), and a shift of the *E*_p_ was noted toward more positive values—all of which is characteristic of an irreversible electrochemical process.

A linear relationship was obtained between *I*_p_ and ν, as shown in the below equation (3):


(3) $$I_{p}(\mu \text{A})=0.018\;\nu \;(\text{mV }{\text{s}}^{-1})+0.34\;(R^{2}=0.993)$$

In contrast, *I*_p_ showed no linear dependence on ν^1/2^, indicating that diffusion does not govern the electron-transfer process. These observations confirm the electrooxidation of ANT on the GCE to be predominantly adsorption-controlled within the investigated scan rate range [19].

**Figure 5:**
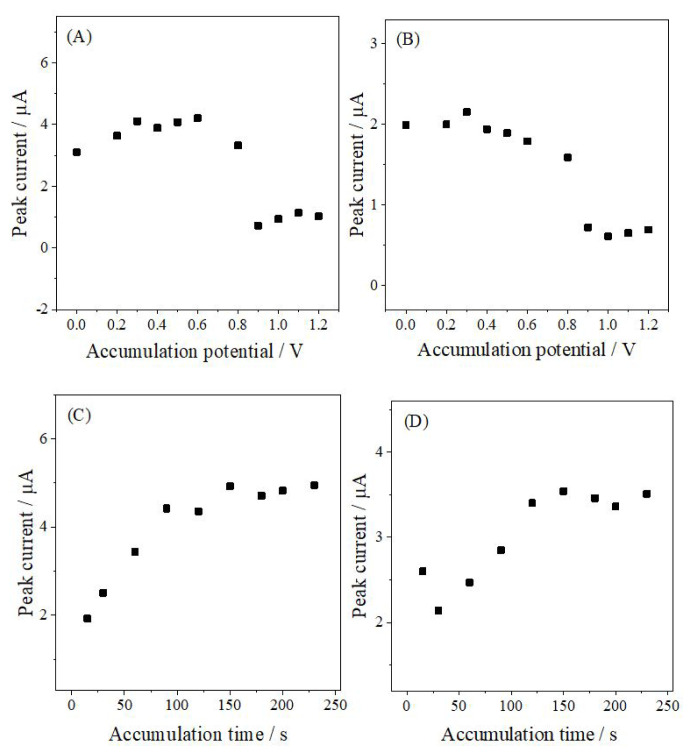
Effect of (A and B) *E*_acc_ and (C and D) *t*_acc_ on the peak current of 1.0 × 10^−5^ M ANT in PB at pH 6.0 using (A and C) AdS-DPV and (B and D) AdS-SWV.

For reliable and sensitive voltammetric determination of ANT, the stripping parameters were (accumulation potential, *E*_acc_, and accumulation time, *t*_acc_) optimized using AdS-DPV and AdS-SWV in 0.1 M PB at pH 6.0 containing 10% acetonitrile. The influence of *E*_acc_ was investigated over the potential range of 0–1.2 V with *t*_acc_ fixed at 60 s. The peak current increased with increasing *E*_acc_ up to 0.6 V for AdS-DPV and 0.3 V for AdS-SWV, after which no further improvement was observed; these values were therefore selected as the optimal *E*_acc_ (Figures 5A and B).

The effect of *t*_acc_ was then examined by varying the *t*_acc_ from 15 to 230 s (Figures 5C and D). The peak current increased initially but approached a plateau at longer times, consistent with surface saturation. Optimal *t*_acc_ values were determined as 150 s for AdS-DPV and 120 s for AdS-SWV, providing well-defined and reproducible peaks. These conditions were used in all subsequent stripping measurements.

### 3.5. Calibration studies

**Figure 6:**
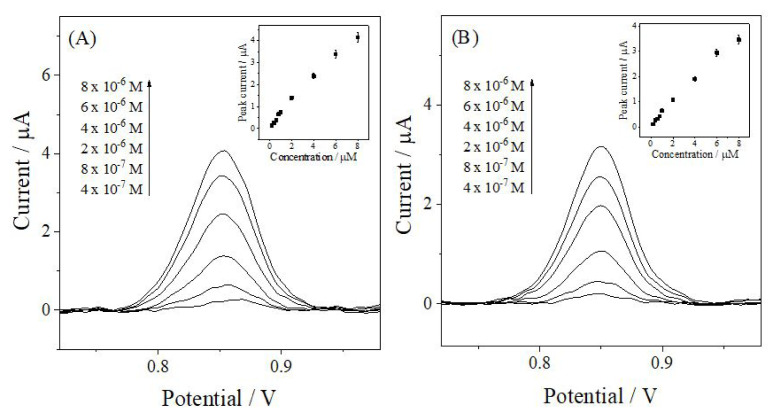
Baseline-corrected (A) AdS-DPV and (B) AdS-SWV voltammograms of ANT at a GCE in PB at pH 6.0. Insets: Calibration plots of peak current versus ANT concentration.

After optimizing the experimental parameters, ANT was quantified using AdS-DPV and AdS-SWV. Calibration curves constructed from peak currents at different ANT concentrations showed linear responses over the 0.2–8.0 μM range (Figure 6). The voltammograms presented in Figure 6 showing selected concentrations illustrate the increase in peak current, although the calibration curves were constructed using all concentration levels within this linear range. Regression data with correlation coefficients above 0.99 (Table 2) confirm the suitability of both techniques for quantitative analysis. The limit of detection (LOD) and limit of quantification (LOQ) were calculated using the expressions 3 *s*/*m* and 10 *s*/*m*, respectively, where *s* is the standard deviation of the analytical response and *m* is the slope of the calibration curve [20]. The resulting values (Table 2) confirm the high sensitivity of the methods.

Method precision was evaluated through intra- and interday studies using 6.0 × 10^−6^ M ANT. Five replicate measurements from three independently prepared solutions yielded %RSD values below 2% for both techniques, indicating good repeatability and intermediate precision.

### 3.6. Determination of ANT in spiked serum samples

The applicability of the AdS-DPV and AdS-SWV to biological matrices was evaluated using protein-free human serum spiked with ANT. No peak was observed in blank serum within the potential window of ANT, confirming the selectivity of both methods. Under the optimized conditions, the calibration plots obtained from the serum samples showed good linearity, covering 0.2–8.0 μM for AdS-DPV and 0.2–6.0 μM for AdS-SWV, with detection limits of 0.0442 μM and 0.0399 μM, respectively (Table 3).

Method precision was assessed through intra- and interday studies using ANT solutions at 6.0 × 10^−6^ M for AdS-DPV and 4.0 × 10^−6^ M for AdS-SWV, yielding RSD values below 1.43% and demonstrating good repeatability. Recovery experiments using serum samples spiked with 2.0 μM ANT provided recoveries of 99.78% for AdS-DPV and 99.52% for AdS-SWV. These results indicate that ANT can be accurately quantified in serum without matrix interference, confirming the suitability of both techniques for biological sample analysis.

### 3.7. Stability studies

The stability of ANT was assessed over four weeks under two storage conditions—room temperature and +4 °C. Stock solutions were stored in the dark, and stability was monitored at 5, 24, 48, 72, and 120 hours as well as after 1, 2, 3, and 4 weeks. At each interval, fresh working solutions were prepared by diluting the stored stock to 4.0 × 10^−6^ M ANT in PB at pH 6.0 containing 10% acetonitrile, and analyzed by CV and SWV.

Under both storage conditions, the peak current values remained essentially constant during the first 2 weeks, indicating that ANT remained stable within this period. Beyond 2 weeks, a gradual decrease in current was observed, suggesting slow degradation of the compound. Overall, ANT stock solutions were stable at room temperature and at +4 °C for up to 2 weeks, indicating adequate short-term stability for routine analytical applications.

### 3.8. Mechanistic investigation

To clarify the possible oxidation mechanism of ANT, 5-nitroisatin and indomethacin were selected as model compounds, being representative of different structural components of the molecule. CV, AdS-DPV, and AdS-SWV measurements were performed under the optimized conditions used for ANT.

No oxidation peak was observed for 5-nitroisatin near 0.8 V, corresponding to the oxidation potential of ANT. This indicates that the isatin-derived part of the molecule does not contribute to the oxidation process. In contrast, indomethacin showed an anodic peak close to that of ANT, suggesting that the two molecules share a common electroactive site. These findings demonstrate that ANT undergoes electrooxidation through the structural fragment it shares with indomethacin rather than through the isatin-related portion of the molecule [21].

The overall mechanistic evaluation indicates that oxidation in the system is strongly biased toward the anthracene core. Anthracene has the highest electron density at the 9,10 positions, facilitating its well-known conversion to anthraquinone via the 9,10-epoxide/diol pathway. Although the 5-nitro substituent increases the electrophilicity of the isatin ring, it does not enhance its susceptibility to oxidation. Instead, by withdrawing electron density, the nitro group effectively passivates the indoline-2-one framework, thereby making oxidation of the isatin moiety less favorable. The resulting C=N-centered oxidation slightly increases the electrophilicity of the imine carbon, but remains a minor pathway and does not dominate the overall reactivity.

**Figure 7:**
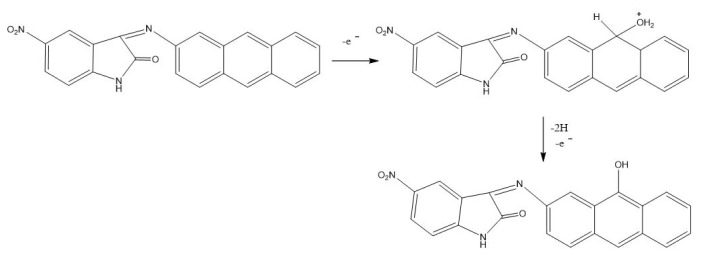
Possible oxidation mechanism of ANT molecule.

Consequently, the 5-nitro group acts as a directing rather than a reactive element, and while it does not participate in the oxidation itself, it shifts the electronic distribution of the molecule, making oxidation at the anthracene 9,10-positions markedly more probable. This electronic steering effect accounts for the preferential oxidation of the anthracene unit over the isatin ring [22] (Figure 7).

A comparison with indomethacin further supports this conclusion. Although indomethacin contains a similar isatin-type core, its oxidation proceeds through a distinct pathway, namely demethylation of the methoxy substituent in the isatin ring, followed by oxidation of the resulting hydroxyl group to a ketone. This contrast demonstrates that the presence of the 5-nitro group in the studied molecule significantly alters the electronic environment and redirects oxidation away from the isatin unit toward the anthracene framework.

## 4. Conclusions

Electrochemical behavior in newly synthesized Schiff base ANT was examined in the present study using CV, DPV, SWV, and their adsorptive stripping variants. The voltammetric response of ANT was strongly affected by solvent composition, pH, and scan rate, with PB at pH 6.0 containing 10% acetonitrile providing the most stable and well-defined oxidation signal. Under these optimized conditions, AdS-DPV and AdS-SWV enabled sensitive and precise quantification of ANT, with low detection limits and high recovery in protein-free human serum. Mechanistic studies using 5-nitroisatin and indomethacin revealed that ANT undergoes oxidation through the anthracene unit rather than the isatin fragment. Overall, the findings enhance the current understanding of the redox behavior of anthracene–isatin Schiff bases and confirm the sensitivity, selectivity, and suitability of the proposed voltammetric methods for analysis in complex matrices.

## Figures and Tables

**Table 1:** Effect of acetonitrile content on the voltammetric response of ANT.

Acetonitrile (%)	CV (μA)	DPV (μA)	SWV (μA)	Peak shape
10	4.3	15.12	8.4	Sharp, well-defined, symmetrical
20	4.4	13.31	8.8	Slightly broadened
30	4.4	9.88	7.1	Broad, less resolved
40	3.8	7.67	6.6	Broad, distorted, poor symmetry

**Table 2:** Calibration characteristics and analytical performance parameters for the voltammetric determination of ANT using AdS-DPV and AdS-SWV.

Parameter	AdS-DPV	AdS-SWV
Peak potential (mV)	852	850
Linear range (μM)	0.2–8.0	0.2–8.0
Slope (μA μM^−1^)	0.52	0.44
Intercept (μA)	0.17	0.11
Correlation coefficient	0.992	0.993
LOD (μM)	3.66 × 10^−2^	4.33 × 10^−2^
LOQ (μM)	1.11 × 10^−1^	1.31 × 10^−1^
Intra-day RSD (%) of peak potential	0.10	0.20
Intra-day RSD (%) of peak current	0.90	0.87
Inter-day RSD (%) of peak potential	0.17	0.29
Inter-day RSD (%) of peak current	1.73	0.99

**Table 3:** Calibration characteristics and analytical performance parameters for ANT determination in spiked human serum using AdS-DPV and AdS-SWV.

Parameter	AdS-DPV	AdS-SWV
Peak potential (mV)	818	818
Linear range (μM)	0.2–8.0	0.2–6.0
Slope (μA μM^−1^)	0.78	0.54
Intercept (μA)	0.28	0.13
Correlation coefficient	0.995	0.992
LOD (μM)	4.42 × 10^−2^	3.99 × 10^−2^
LOQ (μM)	1.34 × 10^−1^	1.21 × 10^−1^
Intra-day RSD (%) of peak potential	0.11	0.11
Intra-day RSD (%) of peak current	0.76	0.90
Inter-day RSD (%) of peak potential	0.17	0.17
Inter-day RSD (%) of peak current	1.04	1.43
Added concentration (μM)	2.000	2.000
Obtained concentration (μM)	1.996	1.990
Average recovered (%)	99.78	99.52
RSD (%) of recovery	1.80	2.38
